# Case-only study of interactions between metabolic enzymes and smoking in colorectal cancer

**DOI:** 10.1186/1471-2407-7-115

**Published:** 2007-06-30

**Authors:** Chunhong Fan, Mingjuan Jin, Kun Chen, Yongjing Zhang, Shuangshuang Zhang, Bing Liu

**Affiliations:** 1 Department of Epidemiology & Health Statistics, School of Public Health, Zhejiang University, Hangzhou 310058, Zhejiang, China; 2 Zhejiang Medical College, Hangzhou 310053, Zhejiang, China

## Abstract

**Background:**

Gene-gene and gene-environment interactions involved in the metabolism of carcinogens may increase the risk of cancer. Our objective was to measure the interactions between common polymorphisms of P450 (CYP1A2, CYP1B1, CYP2E1), GSTM1 and T1, SULT1A1 and cigarette smoking in colorectal cancer (CRC).

**Methods:**

A case-only design was conducted in a Chinese population including 207 patients with sporadic CRC. Unconditional logistic regression analysis was performed adjusting for age, gender, alcohol consumption, and cigarette smoking.

**Results:**

The interaction odds ratio (COR) for the gene-gene interaction between CYP1B1 1294G and SULT1A1 638A allele was 2.68 (95% CI: 1.16–6.26). The results of the gene-environment analyses revealed that an interaction existed between cigarette smoking and the CYP1B1 1294G allele for CRC (COR = 2.62, 95%CI: 1.01–6.72), the COR for the interaction of CYP1B1 1294G and smoking history > 35 pack-years was 3.47 (95%CI: 1.12–10.80). No other significant gene-gene and gene-environment interactions were observed.

**Conclusion:**

Our results showed that the interaction between polymorphisms in CYP1B1 1294G and SULT1A1*2 may play a significant role on CRC in the Chinese population. Also, it is suggested that the association between cigarette smoking and CRC could be differentiated by the CYP1B1 1294G allele.

## Background

Colorectal cancer (CRC) is the third most common malignant tumor, (after lung and breast cancer) worldwide [1]. It has been estimated that there were about 1 million new cases in 2002. China is a nation with relatively low incidence of CRC, being the fifth or sixth most common cause of cancer death in the country. Recently however, the incidence rate of CRC has been increasing rapidly in China [2]. The etiology of CRC is likely to be multifaceted. About 5%–10% of CRC can be attributed to inherited high-penetrance genes; the other 90% may be attributed to diet, lifestyle factors and low-penetrance genes [3,4]. Tobacco smoking is a potential risk factor for CRC [3,5-7]. The burning of tobacco produces a wide variety of carcinogens, including aromatic amines (AAs), heterocyclic amines (HAAs), polycyclic aromatic hydrocarbons (PAHs) and N-nitroso compounds which are metabolized by some Phase I and Phase II metabolic enzymes, such as cytochrome P450s, glutathione S-transferases (GSTs) and sulfotransferases (SULTs) etc. Many of these enzymatic genes are polymorphic and can affect metabolic capabilities, which may result in a differential susceptibility to CRC.

A number of cytochrome P450s, including CYP1A2 and CYP1B1, are involved in the activation of AAs and HAAs [8,9], while CYP2E1 is an ethanol inducible enzyme involved in the activation of N-nitrosamines. A single base C to A change in intron 1 at position 734 downstream was the first identified polymorphism of the *CYP1A2 *gene, and the AA genotype may be associated with increased activity of the enzyme [10]. The Leu432Val amino acid substitution is due to a single C to G base pair change at position 1294 in the *CYP1B1 *gene. This polymorphic variant may cause some altered catalytic specificity with various substrates. In particular, Leu432 variant combined with Arg48, Ser119 and Asn 453 is slightly more active (1.2- to 1.5-fold) than other CYP1B1 enzymes in carcinogen metabolism, such as benzo (a) pyrene [11]. Furthermore, previous studies suggested that the C-to-G transition resulted in an increase of p53 [12]. *Rsa*I polymorphism located in the 5'-flanking region of the *CYP2E1 *was found to affect the transcriptional regulation of the gene, leading to a higher expression level of mRNA among individuals [13].

GSTs belong to a superfamily of detoxification enzymes that facilitate the inactivation of chemical carcinogens and environmental toxic compounds, such as HAAs and PAHs. *GSTM1 *and *GSTT1 *genes are polymorphic and encode the GST-μ and GST-θ enzymes respectively. For both *GSTM1 *and *GSTT1*, the "null" alleles result in a complete loss of enzyme function. SULTs, phase II enzymes with dual functions, are associated with the detoxification and activation of different carcinogens, as well as the regulation of many hormones [14]. SULT1A1 is involved in the bioactivation of HAAs and PAHs [15]. One polymorphism of *SULT1A1 *located in the coding region (638G to A) leads to an Arg213His amino acid change, and the variant A allele (SULT1A1*2) is associated with remarkably reduced sulforansferase activity in platelets compared with the wild-type G allele (SULT1A1*1) [16].

Cigarette smoking and the aforementioned genetic polymorphisms derived from distinct pathways may be associated with CRC. However, minimal information has been previously reported about possible gene-gene and gene-environment interactions. The present study was conducted to explore the role of interactions between cigarette smoking and phase I and phase II metabolic enzymes in CRC, using a nested case-only study in a Chinese Han population cohort.

## Methods

### Study population and sample collection

Participants in this study were selected from a CRC cohort-study population as previously reported [17]. In brief, from May 1989 to April 1990, a prospective cohort study based on a CRC survey was initiated in Jiashan County, Zhejiang province, China. The study population was defined as all residents aged 30 yrs or older in 10 small towns. All subjects were ethnic Han Chinese and residents in Jiashan County. Follow-up for the incidence of cancer in the entire cohort was established by the cancer registry system and CRC reporting system in Jiashan County. 207 individuals, who were diagnosed with CRC before May 2005, comprised the subjects in this analysis. 841 unaffected subjects who were randomly selected from the cohort population were analyzed to examine the assumption of independence between genotypes and cigarette smoking. All subjects were interviewed through a questionnaire, including demographic characteristics, smoking habits, alcohol consumption, and disease history, by professionally trained interviewers. Subjects were considered smokers if they had ever smoked at least one cigarette per day for a period of a year more. The smoking data obtained from study subjects included: the age the subject started smoking, smoking frequency, smoking duration, and inhalation for cigarette smokers. The questions on alcohol consumption included drinking status, average frequency, types of drinks, and average consumption. After informed consent was obtained from each participant, 5 ml of blood was collected and stored at -60°C. The project was approved by the Medical Ethical Committee of the College of Medicine, Zhejiang University.

### Genotyping

Genomic DNA was extracted using the improved salting out procedure. Di-allele -specific-amplification with artificially modified primer (di-ASA-AMP) was used to detect the genotype of *CYP1B1 *C1294G [18]. Two pairs of primers (P1, P2, S1 and S2) are shown in Table 1. The primers of S2 and P2 produced a band representing the C allele (370 bp); the other pair of primer (S1 and P1) produced a band representing the G allele (173 bp). The outer primers (P1 and P2) produced a common band (505 bp).

**Table 1:** Primers and restriction enzymes used for genotyping analyses

Gene	Primers(5'-3'; F = forward, R = reverse)	Restriction enzyme
*CYP1A2 *C734A	F: CCC AGA AGT GGA AAC TGA GA	*Apa*I
	R: GGG TTG AGA TGG AGA CAT TC	
*CYP1B1 *C1294G	P1: TGT CCT GGC CTT CCT TTA TGA	-
	P2: GCC TCT TGC TTC TTA TTG GCA	-
	S1: CCG GGT TAG GCC ACT TGA C	-
	S2: GTC TGT GAA TCA TGA CCG AC	-
*CYP2E1 *C-1019T	F: CCA GTC GAG TCT ACA TTG TCA	*Rsa*I
	R: TTC ATT CTG TCT TCT AAC TGG	
*GSTM1*	F: TTC TGG ATT GTA GCA GAT CA	-
	R: CGC CAT CTT GTG CTA CAT TGC	-
*GSTT1*	F: GCC CTG GCT AGT TGC TGA AG	-
	R: GCA TCT GAT TTG GGG ACC ACA	-
*β*-Globin	F: CAA CTT CAT CCA CGT TCA CC	-
	R: GAA GAA CCA AGG ACA GGT AC	-
*SULT1A1 *G638A	F: GGT TGA GGA GTT GGC TCT GC	*Hha*I
	R: ATG AAC TCC TGG GGG ACG GT	

*GSTM1 *and *GSTT1 *genetic polymorphisms were detected simultaneously in a single assay using a multiplex polymerase chain reaction (PCR) approach [19]. The beta-globin gene co-amplified as an internal positive control, whereas sterile H2O was substituted for genomic DNA and served as a negative control. Positive and negative control samples were analyzed in each experiment. PCR primers are given in Table 1.

The polymerase chain reaction-restriction fragment length polymorphism (PCR-RFLP) method was applied to detect the *CYP1A2 *C734A [20], *CYP2E1 *C-1019T [21], and *SULT1A1 *G638A [22] genotypes. The primers and restriction enzymes used for PCR-PFLP are shown in Table 1. Reliability and validity of the polymorphism analysis were assessed through a repeat analysis using a randomly sampled 10% of the data. No discrepancies were found in the present study.

### Statistical analysis

The χ^2^-goodness-of-fit test was used to test the Hardy-Weinberg equilibrium. For polymorphisms with a low variant allele frequency, the homozygote for the variant allele was combined with the heterozygote, including gene *CYP1B1 *and *SULT1A1*. For certain genes, genotypes were combined on the basis of a known phenotype-genotype relationship, for instance, the combination of *CYP1A2 *734 CC and CA genotypes, *CYP2E1 *-1019 CT and TT genotypes were defined as 'poor metabolizer', while the AA genotype of *CYP1A2 *734 and CC genotype of *CYP2E1 *-1019 were termed as 'extensive metabolizer'.

In order to estimate gene-gene and gene-environment interactions, a case-only design was applied. The case-only design is an efficient way of estimating gene-gene and gene-environment interactions, but it cannot evaluate the main effect of either [23-25]. Such interactions may be obtained in a case-only design if independence between genotypes and between genotypes and environment exposure is assumed. Case-only odds ratios (COR) for the relevant interactions and 95% confidence intervals (95%CI) were estimated via unconditional logistic regression. Possible confounding variables included age, gender, cigarette smoking, and alcohol consumption. Participants with missing values for any of the variables in the regression model were omitted from the analysis.

All p-values are two-sided, and all analyses are carried out using Stata Statistical Software version 8.0.

## Results

### Characteristics of patients with CRC

The characteristics of CRC patients are shown in Table 2. 50.7% of the participants were male and 49.3% female, the mean age being 65.82 years (S.D. = 9.81 years). The observed smoking rate (ever-smoking) was 40.6%, which was lower than the rate of non-smoking (59.4%). Among smokers, 31 presented a smoking history of more than 35 pack-years (36.9%), and 53 presented a smoking history of more than 30 pack-years (63.1%). Excluding one missing response, the observed drinking rate was 28.5%, 35 patients (60.0%) declared moderate drinkers (drink less than 50 g ethanol a day), and 24 patients (40%) declared heavy drinkers.

**Table 2:** Characteristics of CRC study patients

Gender	
Male	105(50.7%)
Female	102(49.3%)
Age (years)	
< 54	28(13.5%)
54–60	40(19.3%)
61–71	70(33.8%)
> 71	69(33.3%)
Cigarette smoking	
Non-smoker	123(59.4%)
Smoker	84(40.6%)
History (pack-years)	
≤ 35	51(24.6%)
> 35	31(15.0%)
No response	2 (1%)
Alcohol consumption	
Non-drinker	147(71.0%)
Moderate drinker (< 50 g ethanol/day)	35(16.9%)
Heavy drinker (≥ 50 g ethanol/day)	24(11.6%)
No response	1 (0.5%)

### The effect of gene-gene interaction on CRC

Table 3 presents the COR for interactions between genotypes in CRC, as determined in the case-only design. All genotype distributions conformed to Hardy-Weinberg equilibrium in this population. A significant interaction was observed between *CYP1B1 *1294G allele and *SULT1A1 *638A allele, the COR being 2.67 (95%CI: 1.14–6.27). However, COR combinations for the other gene-gene interactions were not significant at the five percent level.

**Table 3:** Gene-gene interactions for combinations of metabolic enzymes genotypes in CRC

Genotype	*CYP1A2 *C734A	AA	CC+CA	COR(95%CI)*	*P-*value
*GSTM1*					
Present		37	47	1.00 (Ref.)	0.14
Null		42	78	1.56(0.86–2.82)	
*GSTT1*					
Present		61	92	1.00 (Ref.)	0.59
Null		18	33	1.21(0.62–2.36)	
*SULT1A1*					
*1/*1		68	109	1.00 (Ref.)	0.86
*1/*2,*/*2		11	16	0.93(0.40–2.15)	

	*CYP1B1*C1294G	CC	CG+GG	COR(95%CI)*	*P-*value
*GSTM1*					
Present		50	34	1.00 (Ref.)	0.16
Null		78	40	0.65(0.35–1.19)	
*GSTT1*					
Present		92	69	1.00 (Ref.)	0.08
Null		36	15	0.52(0.25–1.08)	
*SULT1A1*					
*1/*1		117	59	1.00 (Ref.)	0.02
*1/*2,*/*2		13	15	2.67(1.14–6.27)	

	*CYP2E1 *C-1019T	CC	CT+TT	COR(95%CI)*	*P-*value
*GSTM1*					
Present		33	51	1.00 (Ref.)	0.23
Null		58	62	0.70(0.39–1.26)	
*GSTT1*					
Present		66	87	1.00 (Ref.)	0.42
Null		25	26	0.76(0.40–1.47)	
*SULT1A1*					
*1/*1		83	94	1.00 (Ref.)	0.12
*1/*2,*/*2		8	19	2.05(0.82–5.11)	

* Unconditional logistic regression adjusted for gender, age, cigarette smoking and alcohol consumption.

### The effect of gene-environment interaction on CRC

COR for gene-environment interactions between metabolic enzyme genotypes and cigarette smoking is shown in Table 4. Case-only analyses revealed that an interaction existed between cigarette smoking and *CYP1B1 *1294G allele in CRC (COR = 2.62, 95%CI: 1.01–6.72), the COR for the interaction between *CYP1B1 *1294G and smoking history > 35 pack-years was 3.47 (95%CI: 1.12–10.80). No other significant interactions between cigarette smoking and other genotypes were observed.

**Table 4:** Gene-environment interactions for combination of metabolic enzymes and cigarette smoking in CRC

Genotype	Cigarette smoking						
	
	Non-smoker	Smoker	COR (95%CI)*	Smoking history (pack-years)			
				
				≤ 35	COR (95%CI) *	> 35	COR (95%CI)*
*CYP1A2 *C734A							
AA	50	29	1.00(ref.)	17	1.00(ref.)	12	1.00(ref.)
CA+CC	72	53	1.04(0.44–2.50)	33	1.13(0.42~3.01)	18	0.95(0.31~2.93)
*CYP1B1 *C1294G							
CC	82	47	1.00(ref.)	31	1.00(ref.)	16	1.00(ref.)
CG+GG	40	35	2.62(1.01–6.72)^#^	19	1.92(0.72~5.11)	15	3.47(1.12~10.80)^#^
*CYP2E1 *C-1019T							
CC	60	31	1.00(ref.)	18	1.00(ref.)	12	1.00(ref.)
CT+TT	62	51	1.83(0.76–4.40)	32	2.42(0.91~6.46)	18	1.49(0.49~4.53)
*GSTM1*							
Present	52	32	1.00(ref.)	19	1.00(ref.)	12	1.00(ref.)
Null	70	50	1.53(0.62–3.75)	31	1.45(0.55~3.83)	18	1.77(0.58~5.44)
*GSTT1*							
Present	94	59	1.00(ref.)	36	1.00(ref.)	21	1.00(ref.)
Null	28	23	1.32(0.49–3.51)	14	1.41(0.49~4.10)	9	1.79(0.53~6.10)
*SULT1A1*							
*1/*1	106	72	1.00(ref.)	45	1.00(ref.)	25	1.00(ref.)
*1/*2,*2/*2	17	11	0.53(0.16–1.69)	5	0.41(0.11~1.60)	6	0.65(0.16~2.66)

* Unconditional logistic regression adjusted for gender, age and alcohol consumption; # P < 0.05

## Discussion

Epidemiological studies suggested that gene-gene and gene-environment interactions were associated with cancer susceptibility, especially the relationship between metabolic enzyme genes and environmental carcinogens [26-28]. Interactions between carcinogen-metabolizing enzymes and environmental factors may have a marked impact on the population attributable risk for cancer. Therefore, the exploration of the role of gene-gene and gene-environment interactions in low penetrance-genes may elucidate the causation of common sporadic cancers. In the present study, we examined gene-gene and gene-environment interactions among 6 polymorphisms and cigarette smoking through a case-only design.

Few studies have described the association between gene-gene interactions and the risk of CRC to date. Sachse et al. [8] conducted a multicenter case-only study on 490 CRC patients and 593 controls, finding no significant interactions between *GST *and *CYP1A1*. However, in a recent case-control study in China, we have demonstrated that the combinations of *GSTT1 *and *GSTM1 *as well as CYP1A1*2A and *GSTT1 *are significantly associated with CRC [29]. Another study in Taiwan reported that the combination of *CYP2E1 *and *GSTM1 *was associated with breast cancer without the habits of cigarette smoking and alcohol consumption [30]. In contrast, we found no evidence for a significant interaction between *CYP2E1 *and *GSTM1 *as related to CRC in our study population.

It is of particular interest that we found a clear interaction between *CYP1B1 *1294G allele and *SULT1A1 *638A allele in this case-only study. This finding did not support the hypotheses based on in vitro model studies [11,16], suggesting that *SULT1A1*-213Arg (the 638G allele) and *CYP1B1 *432Val (the 1294C allele) enzyme could protect against chemical carcinogens. However, our current knowledge about biotransformation of these carcinogens by these two enzymes is based primarily on in vitro model studies, which may not reflect the complicated situation of carcinogen metabolism in vivo correctly. Bamber et al. [31] reported that a significantly reduced risk of CRC (OR = 0.47; 95% CI: 0.27–0.83) was associated with the *SULT1A1 *638GG genotype in Caucasian subjects under the age of 80. Similar findings have also been reported in the study of other cancers. Dandara et al. [32] revealed that the homozygous *SULT1A1 *638AA genotype was associated with increased risk for oesophageal cancer among smokers. And another study [33] on lung cancer in the Chinese population (805 cases and 809 controls) reported that the variant A allele of *SULT1A1 *was associated with an increased risk of lung cancer (OR, 1.85; 95% CI, 1.44–2.37). Furthermore, these authors showed that the increased risk of lung cancer related to the variant *SULT1A1 *genotypes was more pronounced in younger subjects and limited to smokers. A case-only study on 282 women with breast cancer found that smokers carrying the *SULT1A1 *638 A allele had a two-fold increase in risk compared to non-smokers carrying the *SULT1A1 *638GG genotype (OR = 2.55, 95%CI: 1.21–5.36), and smokers carrying the *CYP1B1 *1294G allele also had a higher risk of breast cancer than non-smokers carrying the 1294CC genotype (OR = 2.32, 95%CI: 1.00–5.38) [34]. These findings indicated that SULT1A1 and CYP1B1, among many other metabolic pathways, may not be of importance in the metabolic activation of these carcinogens in vivo.

However, other possibilities may also exist for the explanation of our results. Accumulating evidence suggested that CRC is a hormone dependent tumor. For instance, epidemiological studies found that a correlation existed between breast cancer and colorectal cancer in women [35,36]. A meta-analysis of seven cohort studies demonstrated that a weak association existed between breast cancer and the subsequent risk of colorectal cancer (pooled relative risk (RR) = 1.15; 95% CI = 0.99–1.31], and pooled results from five cohort studies showed that the risk of breast cancer after colorectal cancer was similar (pooled RR = 1.10; 95% CI = 1.03–1.17) [37]. In addition, a meta-analysis by Schoen et al. [38] indicated that women with a history of breast, endometrial, or ovarian cancer are at a statistically significant increased risk for subsequent colorectal cancer. The study of English et al. [39] indicated that the loss of estrogen inactivation may be an important mechanism in the pathogenesis of colonic cancer. The normal colonic mucosa has a high level of 17beta-hydroxysteroid dehydrogenase (17beta-HSD)-mediated estradiol metabolism (inactivation of estradiol to estrone), while the enzyme activity of 17beta-HSD was significantly decreased in the tumor [40]. Taken together, these findings suggest that the oestrogen metabolism in vivo might be associated with increased risk of CRC. CYP1B1 and SULT1A1 are all important estrogen-metabolizing enzymes. CYP1B1 is responsible for catalyzing the formation of 4-hydroxy-estradiol, while SULT1A1 is involved in the inactivation of these carcinogens. The variant of *CYP1B1 *displayed increased catalytic efficiency compared to the wild-type enzyme, and may be associated with significant changes in oestrogen metabolism. The combination of the *CYP1B1 *1294G allele and the *SULT1A1 *638A allele with modified ability to metabolize estrogens could increase the level of estrogen (i.e., 4-hydroxy-estradiol), which may finally result in a differential susceptibility to CRC.

Our gene and environment combined analyses suggested that a significant interaction, which may increase the risk for CRC, exists between the *CYP1B1 *1294G allele and cigarette smoking. This finding also did not support the hypothesis based on in vitro model studies [11], and no reports have described the association between this gene-environment interaction and CRC risk to date. However, the same gene-environment interaction was reported in studies on other cancers, the results of which were similar to ours. Liang et al. [18] found that *CYP1B1 *432Val polymorphism may modulate individual susceptibility to lung cancer among smokers in the Chinese population (OR: 2.78, 95%CI: 1.46–5.29). A case-only study on breast cancer also reported that the combination of cigarette smoking and *CYP1B1 *1294G allele was associated with a higher risk of breast cancer [34]. Taken together, in contrast to the hypothesis based on the in vitro model studies, these findings suggest that the 1294CC genotype may protect against chemical carcinogens related to certain human cancers, such as PAHs. In addition, whether tobacco contains numerous carcinogens and compound(s) that assuredly cause human CRC still remains to be seen. Further studies are needed to address these possibilities.

A few case-control studies have been published with regard to the combination of *GSTT1/GSTM1 *and cigarette smoking in association with CRC. A study of 952 rectal cancer cases and 1205 controls in the United States found that a borderline significant interaction existed between the *GSTM1 *null allele and cigarette smoking [41]. Another cohort study on smoking with a nested case-only design also found a borderline significant interaction between *GSTM1 *null and cigarette smoking [42]. However, two studies indicated that no significant interactions between *GSTT1/GSTM1 *and cigarette smoking were observed [43,44], the results of which were similar to ours. Larger studies are needed to explore the interactions between the susceptible genes and smoking and to identify the underlying mechanisms of CRC.

One of the limitations of the case-only design is that, in order to obtain gene-gene and gene-environment interactions, independence between genotypes and between genotypes and environment exposure must be assumed. In this study, we validated this independence assumption through the analysis of a relatively large number of healthy subjects (n = 841, 62.55 ± 10.85 years) randomly selected from the same cohort population. Another limitation of the current study is that selection bias might occur from all patients being cancer survivors at the time of analysis. However, since we used incident cases recruited from the cohort population, our findings are unlikely to be attributable to selection bias.

## Conclusion

Our results showed that the interaction between polymorphisms in *CYP1B1 *1294G and SULT1A1*2 play a significant role on CRC in Chinese people, and the association between cigarette smoking and CRC can be differentiated by the *CYP1B1 *1294G allele. However, these preliminary exploratory results should be confirmed in larger studies.

## Competing interests

The author(s) declare that they have no competing interests.

## Authors' contributions

CF participated in the design of the study, SNP genotyping, and drafted the manuscript. KC conceived of the study, and participated in its design and coordination. MJ, YZ and SZ participated in data collection and performed the statistical analysis. BL participated in SNP genotyping. All authors read and approved the final manuscript.

## Pre-publication history

The pre-publication history for this paper can be accessed here:


